# Differential expression of miR-195-5p in collapse of steroid-induced osteonecrosis of the femoral head

**DOI:** 10.18632/oncotarget.17333

**Published:** 2017-04-21

**Authors:** Pengfei Li, Pei Zhai, Zengjie Ye, Peng Deng, Yueguang Fan, Yirong Zeng, Zhihui Pang, Jianchun Zeng, Jie Li, Wenjun Feng

**Affiliations:** ^1^ Guangzhou University of Chinese Medicine, Guangzhou, Guangdong Province, China; ^2^ Yale University, New Haven, Connecticut, USA; ^3^ Department of Orthopedics, The First Affiliated Hospital of Guangzhou University of Chinese Medicine, Guangzhou, Guangdong Province, China

**Keywords:** femoral head collapse, miR-195-5p, downregulated, osteonecrosis

## Abstract

**Background:**

Femoral head collapse is a key reference point for determining a treatment regimen of femoral head osteonecrosis. However, there are no effective preventive measures and the efficacy of hip-preserving surgery is unsatisfactory due to the unclear mechanism of collapse. This study aimed to identify and validate miRNAs differentially expressed in collapse and non-collapse areas of the osteonecrotic femoral head, and to predict the target genes and pathways of these miRNAs.

**Results:**

Nine samples passed the quality control test. A total of 2085 differentially expressed miRNAs were detected, among which 433 miRNAs showed differential expression in the T1 group compared to the W1 group; 344 miRNAs showed differential expression in the T2 group compared to the W2 group; 107 miRNAs showed differential expression in the T3 group compared to the W3 group. After combining data from all three patients, 10 miRNAs showed differential expression in the collapse area (T1+T2+T3) compared to the non-collapse area (W1+W2+W3). Compared to the normal area, has-miR-195-5p showed the most significant downregulation. Expression results from RT-PCR revealed that the expression of hsa-miR-195-5p in the collapse area (T1+T2+T3) was significantly lower than that in the non-collapse area (W1+W2+W3) and normal area (Z1+Z2+Z3). 157 genes were perdicted as the target gene of hsa-miR-195-5p.

**Materials and Methods:**

Femoral heads of three patients (2 males and 1 female) treated by total hip arthroplasty surgery for steroid-induced femoral head osteonecrosis were selected based on inclusion and exclusion criteria. Bone tissue samples were obtained from the collapse area (T), non-collapse area (W), and normal area (Z) according to the anatomical structure of osteonecrotic femoral heads. Total RNA was extracted from the samples and the microarray chip was scanned. miRNAs showing differential expressions of more than 1.5-fold were selected and was validated by RT-PCR. TargetScan, mirBase and miRanda bioinformatics software was used to predict target genes and identify possible pathways involving these genes.

**Conclusions:**

miR-195-5p showed the most significant difference in the collapse area of osteonecrotic femoral heads, suggesting that collapse may be related to the downregulation of miR-195-5p.

## INTRODUCTION

Steroid-induced osteonecrosis of the femoral head is a disabling disease, particularly after the 2003 SARS(severe acute respiratory syndrome) outbreak in China, but the mechanism of whom is not completely understood [1]. Approximately 20,000–30,000 people are diagnosed with osteonecrosis in the United States each year [2]; the morbidity rate of non-traumatic femoral head osteonecrosis in Japan was 1.91/100,000 per year [3]. The natural course of femoral head osteonecrosis is necrosis, followed by collapse and osteoarthritis. Once collapse occurs, osteoarthritis and joint replacement is inevitable [4, 5]. Therefore, collapse must be treated surgically for femoral head osteonecrosis, and preventing collapse has been the focus of non-joint replacement therapy [6–14]. Current studies of collapse are typically performed from the biological, biomechanical, or mechanobiological perspective [15–19]; however, the mechanism of collapse is unclear, leading to insufficient effective preventive measures and unsatisfactory efficacy of hip-preserving surgery. As the number of studies of miRNA in orthopedics has increased, specific miRNAs involved in femoral head osteonecrosis have been identified [20–24]. However, the developmental course of femoral head osteonecrosis is a dynamic process. Whether miRNA is differentially expressed in different states of osteonecrotic femoral head (non-collapse vs. collapse) remains unclear. Studies of differential miRNA expression may increase the understanding of the biological mechanisms underlying collapse. This study aimed to identify miRNAs differentially expressed in collapse and non-collapse areas of the osteonecrotic femoral head and validate these miRNAs using reverse transcription-polymerase chain reaction (RT-PCR) assays, and eventually predict the target genes and pathways of these miRNAs.

## RESULTS

### Quality control and results of differentially expressed miRNAs

**Figure 1:**
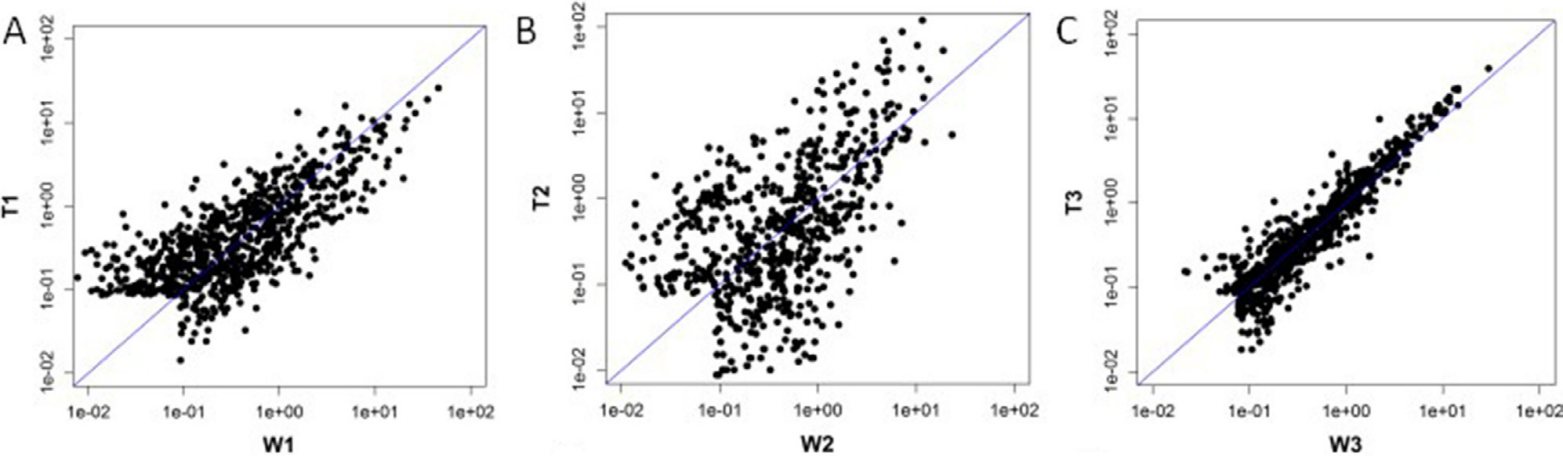
Scatter Plot: T1 vs W1 was showed 1A, T2 vs W2 was showed 1B,T3 vs W3 was showed 1C

The 9 selected samples passed the quality control test. A total of 2085 differentially expressed miRNAs were detected in the 9 samples. Among the detected miRNAs, 433 miRNAs showed more than 1.5-fold differential expression in the T1 group compared to the W1 group (247 miRNAs were up-regulated and 186 miRNA were down-regulated); 344 miRNAs were differentially expressed by more than two-fold in the T2 group compared to the W2 group (225 miRNAs were up-regulated and 119 miRNAs were down-regulated); 107 miRNAs showed differential expression by more than two-fold in the T3 group compared to the W3 group (51 miRNAs were up-regulated and 56 miRNAs were down-regulated) (Figure 1). Combining the data from all three patients, 10 miRNA were differentially expressed by more than two-fold in the collapse area (T1+T2+T3) compared to the non-collapse area (W1+W2+W3) (8 miRNAs were up-regulated: hsa-miR-4472, hsa-miR-4306. hsa-miR-4747-5p, hsa-miR-4441, hsa-miR-4709-3p, ebv-miR-BHRF1-2-3p, hsa-miR-585-3p, and hsa-miR-5572, while 2 miRNAs were down-regulated: hsa-miR-195-5p and hsa-miR-645). Compared to the normal region, has-miR-195-5p showed the most significant down-regulation (Tables 1, 2, 3).

**Table 1:** Compared with the W1, the number of differential expression of miRNAs (fold change > 1.5) in T1

miRNA	Fold change	ForeGround			ForeGround-BackGround			Normalized		
	T1 vs W1	W1	T1	Z1	W1	T1	Z1	W1	T1	Z1
hsa-miR-4472	2.63610029	141.5	250.5	137	71	203	31.5	0.218461538	0.575886525	0.101777060
hsa-miR-4306	2.731554991	207	444	184	134	397	70	0.412307692	1.126241135	0.226171244
hsa-miR-4747-5p	2.461107298	252	542	448.5	186	496.5	358.5	0.572307692	1.408510638	1.158319871
hsa-miR-4441	3.793744913	105	173.5	106	30.5	125.5	12	0.093846154	0.356028369	0.038772213
hsa-miR-4709-3p	3.75177305	132.5	314.5	301	65	264.5	208	0.2	0.75035461	0.672051696
ebv-miR-BHRF1-2-3p	5.163120567	130	395	171	62.5	350	77	0.192307692	0.992907801	0.248788368
hsa-miR-585-3p	6.490057016	120.5	406	98.5	51	359	3.5	0.156923077	1.018439716	0.011308562
hsa-miR-5572	4.292524125	100	186.5	148.5	30.5	142	51	0.093846154	0.402836879	0.164781906
hsa-miR-195-5p	0.111243115	2628	355.5	4311	2561	309	4215	7.88	0.876595745	13.61873990
hsa-miR-645	0.446777267	296.5	157	95.5	227	110	2.5	0.698461538	0.312056738	0.008077544

**Table 2:** Compared with the W2, the number of differential expression of miRNAs (fold change > 1.5) in T2

miRNA	Fold change	ForeGround			ForeGround-BackGround			Normalized		
	T2 vs W2	W2	T2	Z2	W2	T2	Z2	W2	T2	Z2
hsa-miR-4472	16.93309671	144	1958.5	341.5	99	1841	300.5	0.273858921	4.637279597	0.815468114
hsa-miR-4306	5.720926398	310	1792	160	263.5	1655.5	116.5	0.728907331	4.170025189	0.316146540
hsa-miR-4747-5p	3.317858073	653	2359	675	609	2219	635.5	1.684647303	5.589420655	1.724559023
hsa-miR-4441	7.760619418	68.5	312	158.5	22	187.5	116.5	0.060857538	0.472292191	0.316146540
hsa-miR-4709-3p	7.548223518	84	440	211.5	38	315	170	0.105117566	0.793450882	0.461329715
ebv-miR-BHRF1-2-3p	5.321105359	196.5	1028.5	203	153.5	897	164.5	0.42461964	2.259445844	0.446404342
hsa-miR-585-3p	5.103435283	285.5	1472	395.5	239	1339.5	353.5	0.661134163	3.374055416	0.959294437
hsa-miR-5572	2.181596348	68	176.5	53.5	24	57.5	15.5	0.066390041	0.144836272	0.042062415
hsa-miR-195-5p	0.057324608	870	183	1330	826	52	1290.5	2.284923928	0.130982368	3.502035278
hsa-miR-645	0.01983833	275.5	131	81	229.5	5	39.5	0.634854772	0.012594458	0.107191316

**Table 3:** Compared with the W3, the number of differential expression of miRNAs (fold change >1.5) in T3

miRNA	Fold change	ForeGround			ForeGround-BackGround			Normalized		
	T3 vs W3	W3	T3	Z3	W3	T3	Z3	W3	T3	Z3
hsa-miR-4472	2.265840182	94.5	149.5	107.5	50.5	102	25	0.127525253	0.288951841	0.075872534
hsa-miR-4306	5.28542706	330	1374	456	282.5	1331	376.5	0.713383838	3.770538244	1.142640364
hsa-miR-4747-5p	2.097163586	1797	3320	366	1754	3279	287	4.429292929	9.288951841	0.871016692
hsa-miR-4441	3.5898017	82.5	174	123.5	40	128	49.5	0.101010101	0.362606232	0.150227618
hsa-miR-4709-3p	5.375354108	77.5	215.5	696	36	172.5	620.5	0.090909091	0.488668555	1.883156297
ebv-miR-BHRF1-2-3p	2.243626062	198.5	351.5	131.5	157	314	60	0.396464646	0.889518414	0.182094082
hsa-miR-585-3p	3.089906068	103	199	88.5	57	157	10.5	0.143939394	0.444759207	0.031866464
hsa-miR-5572	4.599433428	66.5	121	326	20	82	252	0.050505051	0.232294618	0.764795144
hsa-miR-195-5p	0.487103027	384.5	188.5	1268.5	342	148.5	1195.5	0.863636364	0.420679887	3.628224583
hsa-miR-645	0.46888279	170	98	154	128	53.5	78	0.323232323	0.151558074	0.236722307

### RT-PCR results of hsa-miR-195-5p

**Figure 2:**
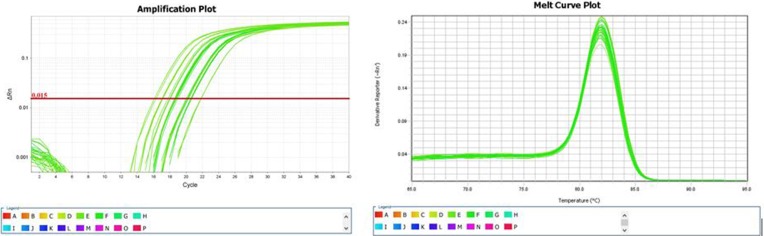
Amplification and melting curves of hsa-miR-195-5p

The following U6 primers were used for RT-PCR:F: 5′GCTTCGGCAGCACATAT ACTAAAAT3′, R: 5′CGCTTCACGAATTTGCGTGT CAT3′. The primer sequences of hsa-miR-195-5p were: GSP: 5′GGGGTAGCAGCACAGAAAT3′, R: 5′CAGTGCGTGTCGTGGAGT3′(GSP is a specific primer for miRNA, while R is a primer that matches the RT primer). The RT-PCR expression level of hsa-miR-195-5p was significantly lower in the collapse area (T1 T2 T3)than in the non-collapse area (W1 W2 W3) and normal area (Z1 Z2 Z3). (Table 4) Comparison of the results from RT-PCR and the microarray data also indicated that hsa-miR-195-5p expression was downregulated. Amplification and melting curves were obtained (Figure 2).

**Table 4:** The RT-PCR results of hsa-miR-195-5p

Sample number	U6Ct	(hsa-miR-195-5p)Ct	(hsa-miR-195-5p)Ct-U6Ct	2^−ΔΔCT^
T1	15.894	20.228	4.333	1.00
W1	15.867	17.137	1.270	8.36
Z1	15.521	16.211	0.689	12.50
T2	15.774	21.837	6.063	0.30
W2	15.871	17.946	2.074	4.78
Z2	15.555	18.715	3.160	2.25
T3	15.449	20.638	5.189	0.55
W3	15.752	20.307	4.555	0.86
Z3	15.848	18.838	2.990	2.54

### Target genes, network, GO analysis and KEGG pathway analysis of hsa-miR-195-5p

**Figure 3:**
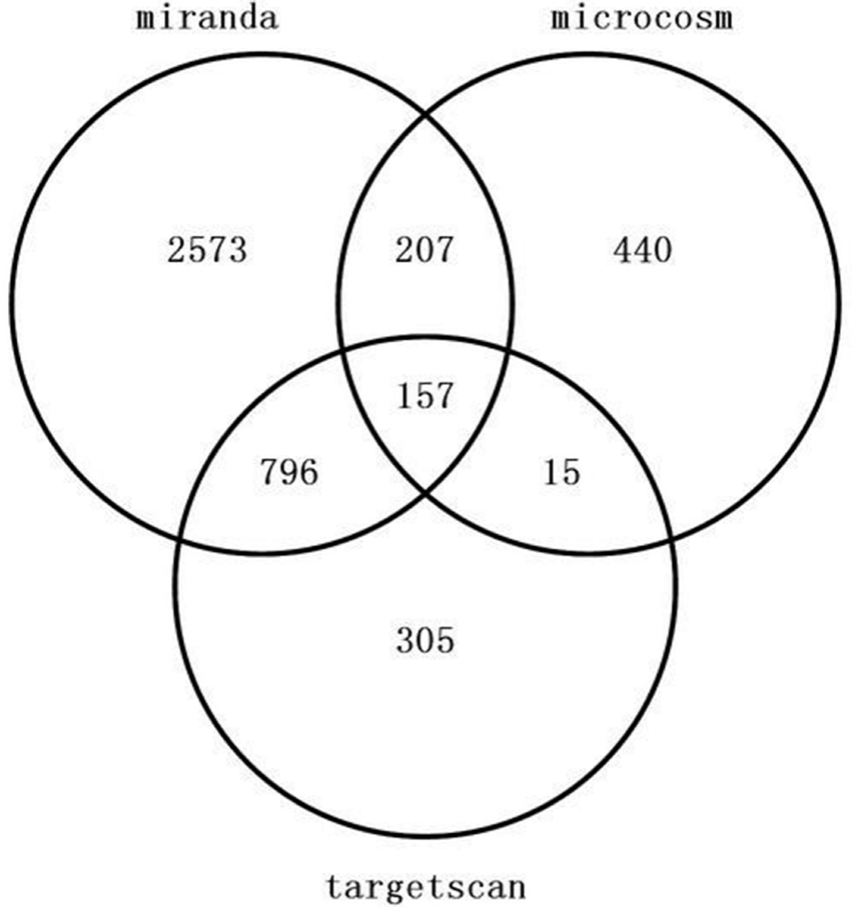
Venn diagram of target gene of hsa-miR-195-5p

**Figure 4:**
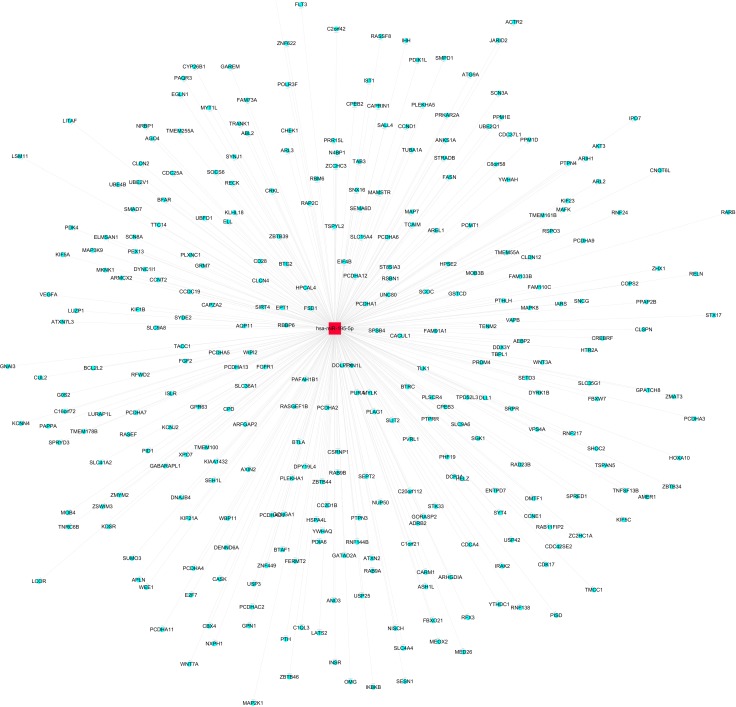
Network of target gene of hsa-miR-195-5p

**Figure 5:**
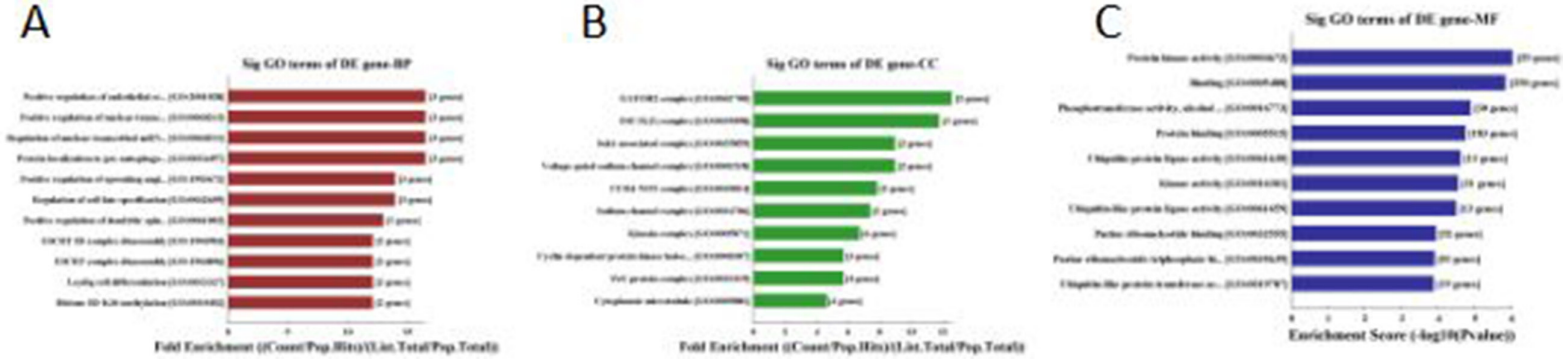
GO analysis of hsa-miR-195-5p: 5A was biological process, 5B was cellular component and 5C was molecular function

**Figure 6:**
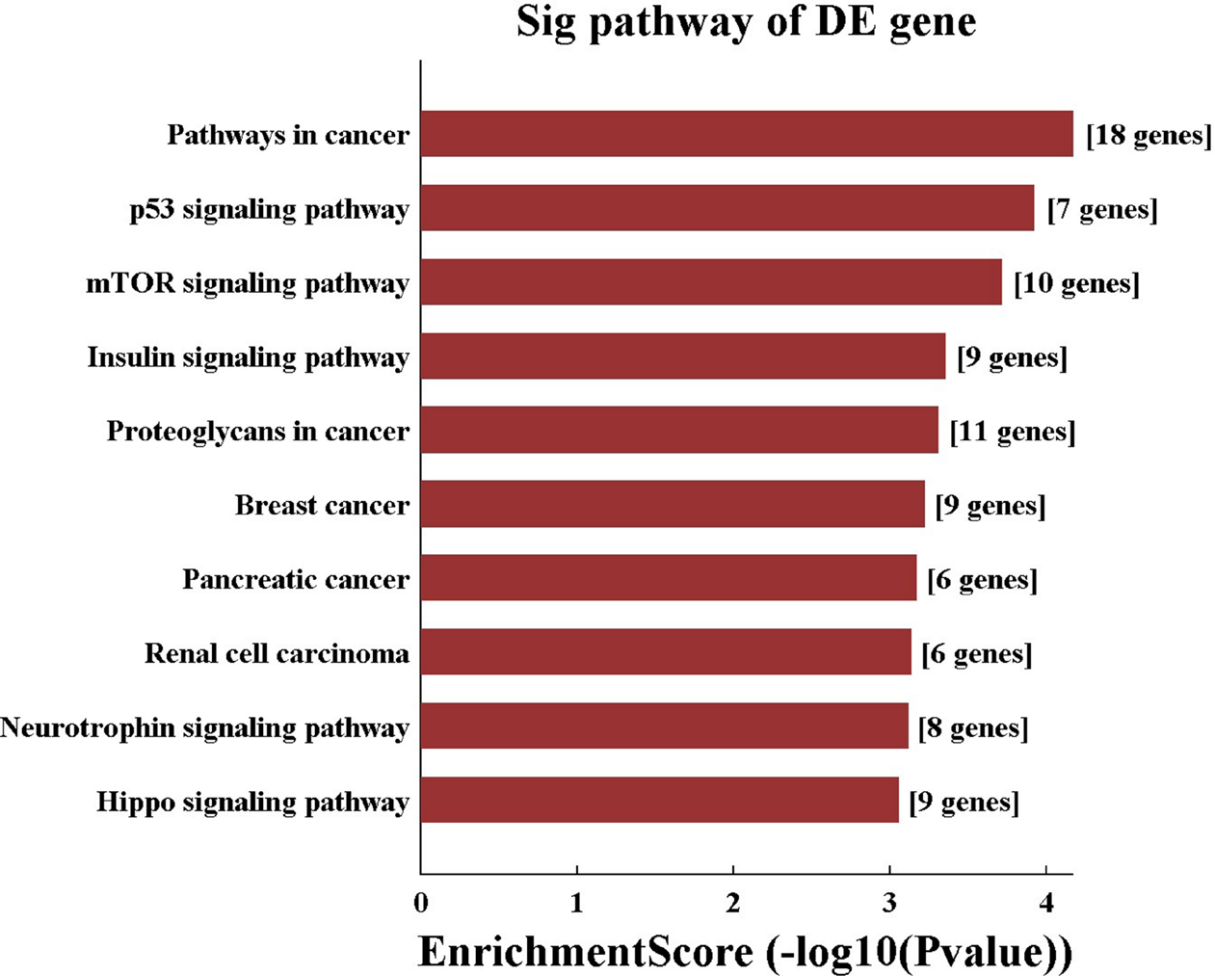
KEGG pathway analysis of hsa-miR-195-5p

157 genes were perdicted as the target gene of hsa-miR-195-5p (Figures 3, 4). GO analysis and KEGG Pathway analysis were showed that pathway in cancer is significant (Figures 5, 6).

## DISCUSSION

Previous studies have suggested that collapse occurs because of an imbalance between bone resorption and bone formation after femoral head osteonecrosis, resulting in femoral heads with reduced structural strength that are unable to bear external forces. The studies have also suggested that mechanical support and adjustment of bone cell metabolism can prevent collapse [25]. To comprehensively understand the biological behavior of osteocytes, an increasing number of studies have focused on the differential expression of miRNAs associated with osteonecrosis of the femoral head [23–24, 26–28]. We evaluated whether the decrease in structural strength was associated with the differential expression of relevant miRNAs, which may lead to faster apoptosis and an increased imbalance with bone metabolism. We found that in the osteonecrotic region, hsa-miR-195-5p was down-regulated in the collapse area compared to in the non-collapsed area. This is the first study examining the relationship between miRNA and femoral head collapse.

The anatomical structure of osteonecrotic femoral head has four layers: cartilage, osteonecrotic area, sclerotic area, and normal area. Recent studies have proposed that the decrease in structural strength is correlated with sclerotic area and osteonecrotic edge, enabling collapse prediction [16, 19]. During the pathological process, collapse of the osteonecrotic femoral head occurs because of continuous spreading of trabecular fracture, which is not a sudden process, and thus the osteonecrotic area includes the collapse area and non-collapse area. Femoral head collapse originates from subchondral fracture. Therefore, in this study, subchondral fracture in the osteonecrotic femoral head was defined as the collapse area, while the area in the osteonecrotic femoral head without subchondral fracture was defined as the non-collapse area. Non-collapse and collapse are different pathological stages of osteonecrotic femoral head. We not only developed a method for predicting the risk of collapse, but also provide a new insight into preventing collapse using gene editing techniques.

It has been reported that miR-195-5p is a tumor-suppressor gene that mainly suppresses cell proliferation and invasion and is down regulated in cancer patients [29–34]. This gene inhibits hepatocellular carcinoma by regulating PHF19 [30], regulates rectal cancer through CDK8, regulates osteosarcoma by inhibiting NKD1 [33], regulates prostate cancer through Fra-1 or RPS6KB1 [34]. Based on these results, we hypothesized that the mechanism of collapse involves down-regulation of miR-195-5p, which disrupts the proliferation of normal osteoblasts and accelerates cell apoptosis. This leads to a further decrease in structural strength within the femoral head and failure to support external forces, eventually resulting in collapse. Future studies should examine methods of increasing the expression of miR-195-5p to treat cancer and the collapse of femoral head osteonecrosis.

The limitations of this study were as follows: firstly, small sample size, but with reproducibility; secondly, target genes and pathways were only predicted and require further validation; In addition, whether collapse leads to miRNA changes or miRNA changes lead to collapse remains unknown. These factors exhibit a mutually causal relationship. In future studies, we will evaluate the genetic pathways of the collapse-associated differential miRNAs and gene editing technology to prevent and treat collapse.

## MATERIALS AND METHODS

### Material collection

Three patients were randomly selected from the hospital system in our department. Inclusion criteria were as follows: 1. diagnosed with steroid-induced femoral head osteonecrosis (a clear history of hormone use); 2. diagnosed with ACRO III/IV stage based on X-ray by 3 doctors; and 3. the surgeon and patient decided to perform the total hip joint replacement surgery. Exclusion criteria were as follows: 1. The cause of femoral head osteonecrosis was not clear or involved multiple factors; 2. Severe liver and kidney failure in which the patient could not tolerate surgery; And 3. Surgical strategy in which patient consent was not obtained. Informed consent was obtained from all patients and the ethical review committee of our hospital. According to the inclusion and exclusion criteria, three patients were selected. There were 2 males, one of whom at the age of 30 years used hormones to treat thrombocytopenic purpura, while the other, at the age of 46 years, used hormones to treat skin disease. At the age of 20 years, the female patient used hormones to treat systemic lupus erythematosus.

### Sample selection

**Figure 7:**
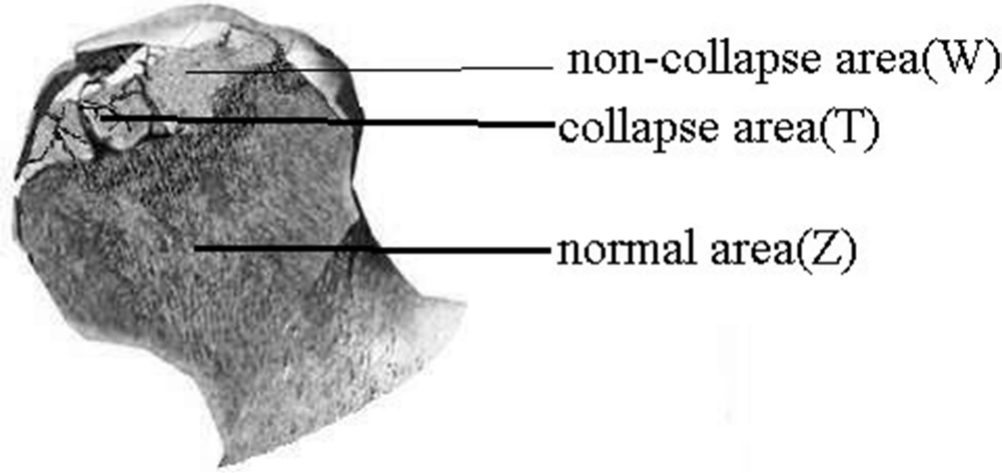
Bone tissues were taken from normal area, collapse area, and non-collapse area of femoral heads

After surgery, femoral heads were removed and cleaned with saline. Three bone tissues (normal area, collapse area, and non-collapse area) were taken from femoral heads (Figure 7). Two doctors identified these tissues, and a total of 9 samples were obtained (T1 T2 T3, W1 W2 W3, and Z1 Z2 Z3). Bone tissues were immediately stored in liquid nitrogen and ground into powder (≥ 100 mg) in liquid nitrogen. The powder was transferred to an microcentrifuge tube, mixed with 1 mL Trizol, and stored at **−**70°C until use.

### Total RNA extraction, RNA labeling, hybridization and microarray chip scanning [23]

#### Total RNA extraction and quality control

Total RNA was extracted from tissue cells using Trizol Reagent (Invitrogen life technologies) according to the manufacturer's instructions; total RNA was extracted from whole blood using TRI reagent BD (MRCgene, TB-126). Total RNA was extracted from plasma and serum exosomes using TRIzol LS. RNAsey Mini Kit (QIAGEN) was used for RNA purification; NanoDrop ND-1000 was employed for measurement of RNA concentration after purification. RNA integrity was detected by electrophoresis.

### RNA labeling and hybridization using the exiqon method [23]

After extraction, RNA samples underwent quality control and miRNA was labeled using miRCURYTM Array Power Labeling kit (Cat #208032-A, Exiqon). The detailed steps are described as follows:One microgram RNA sample was added to 2 μL water, and then 1 μL CIP buffer and CIP enzyme (Exiqon) were added to the RNA sample solution, and the mixture solution was incubated at 37°C for 30 min. The reaction in the sample solution was terminated at 95°C for 5 min, and then 3 μL labeling buffer, 1.5μL fluorescent label (Hy3^™^), 2.0 μL DMSO, and 2.0 μL labeling enzyme were added and the solution was incubated at 16°C for 1 h. The reaction in the samples was terminated at 65°C for 15 min.

After labeling, the sample was hybridized with miRCURYTM LNA Array (v.18.0) (Exiqon), and the subsequent procedures were conducted according to Exiqon's experimental methods. The hybridization system used was the Nimblegen system (Nimblegen Systems Inc., Addison, WI, USA). The microarray chip was rinsed with Wash buffer kit (Exiqon) after hybridization.

### The microarray chip was scanned by the axon GenePix 4000B Microarray Scanner [23]

**Figure 8:**
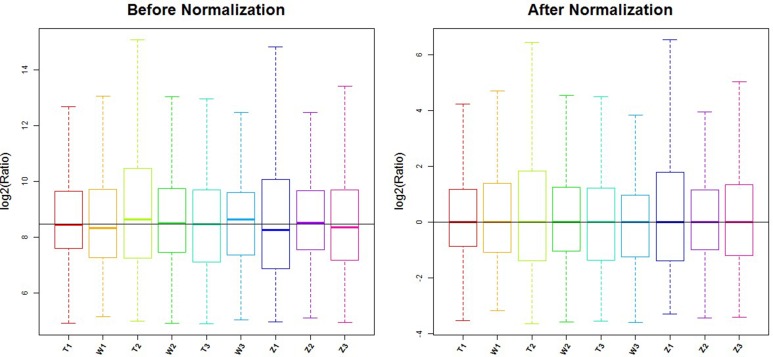
Box plot: after normalization, the median line of 9 samples were equal

GenePix Pro V6.0 was utilized to read the microarray scan images and the probe signals were selected. The median was taken and combined for the same probes. Probes >= 30.0 for the samples were kept and the median was standardized in all microarray chips. (Figure 8) miRNAs showing differential expressions of more than 1.5-fold were selected [24].

### Verification of significantly differentially expressed miR-195-5p by RT-PCR

cDNA synthesis of RNA samples [23]. 20 μL of RT reaction mixture was prepared, then all cDNA samples were separately set up in the RT-PCR System (Applied Biosystems); The solution was mixed up to 8 μL and centrifuged at 5000 rpm for a short period.

The mixture was added to each well of a 384-well PCR plate, and then 2 μL corresponding cDNA was added to each well. The plate was sealed with sealing film and centrifuged. The 384-well PCR plate was placed in the RT-PCR amplification instrument to carry out the PCR reaction. U6 (internal reference) and all parameters were conducted.

The RT-PCR reaction was performed for the target miRNA in all samples and the internal reference gene (U6) separately. In Table 1, data analysis was performed using 2^−ΔΔCT^ method. SPSS 20.0 (SPSS Inc., Chicago, Ill., USA) was employed for statistical analysis of the data.

### Predication of target gene of miR-195-5p

TargetScan, mirBase and miRanda bioinformatics software were used to predict target genes. Gene Ontology(GO, http://www.geneontology.org/) analysis and Kyoto Encyclopedia of Genes and Genomes(KEGG, www.genome.jp/kegg/) pathway analysis were done respectively. The GO project covers three domains: Biological Process, Cellular Component and Molecular Function. Fisher's exact test is used to find if there is more overlap between the differential expression (DE) list and the GO annotation list than would be expected by chance. The *p*-value denotes the significance of GO terms enrichment in the DE genes. The lower the *p*-value, the more significant the GO Term (*p*-value <= 0.05 ). Pathway analysis is a functional analysis mapping genes to KEGG pathways. The *p*-value denotes the significance of the Pathway correlated to the conditions. Lower the *p*-value, more significant is the Pathway(*p*-value cut-off is 0.05).
